# Recovery of chloroplast genomes from medieval millet grains excavated from the Areni-1 cave in southern Armenia

**DOI:** 10.1038/s41598-022-17931-4

**Published:** 2022-09-07

**Authors:** Stephen M. Richards, Leiting Li, James Breen, Nelli Hovhannisyan, Oscar Estrada, Boris Gasparyan, Matthew Gilliham, Alexia Smith, Alan Cooper, Heng Zhang

**Affiliations:** 1 School of Biological Science, The University of Adelaide, Adelaide, Australia; 2 National Key Laboratory of Plant Molecular Genetics, Shanghai Center for Plant Stress Biology, CAS Center for Excellence in Molecular Plant Sciences, Chinese Academy of Sciences, Shanghai, China; 3 Yerevan State University, Yerevan, Armenia; 4 Institute of Archaeology and Ethnography, National Academy of Sciences of the Republic of Armenia, Yerevan, Armenia; 5 Waite Research Institute and School of Agriculture, Food, and Wine, ARC Centre of Excellence in Plant Energy Biology, The University of Adelaide, Waite Campus, Glen Osmond, Australia; 6 Department of Anthropology, University of Connecticut, Connecticut, USA; 7 BlueSky Genetics, Ashton, SA Australia; 8 South Australian Museum, Adelaide, SA Australia; 9 Present Address: Telethon Kids Institute, Australian National University, Canberra, Australia; 10 Present Address: Grupo de Agrobiotecnología, Instituto de Biología, Universidad de Antioquia, Medellín, Colombia

**Keywords:** Plant genetics, Archaeology

## Abstract

*Panicum miliaceum* L. was domesticated in northern China at least 7000 years ago and was subsequentially adopted in many areas throughout Eurasia. One such locale is Areni-1 an archaeological cave site in Southern Armenia, where vast quantities archaeobotanical material were well preserved via desiccation. The rich botanical material found at Areni-1 includes *P. miliaceum* grains that were identified morphologically and^14^C dated to the medieval period (873 ± 36 CE and 1118 ± 35 CE). To investigate the demographic and evolutionary history of the Areni-1 millet, we used ancient DNA extraction, hybridization capture enrichment, and high throughput sequencing to assemble three chloroplast genomes from the medieval grains and then compared these sequences to 50 modern *P. miliaceum* chloroplast genomes. Overall, the chloroplast genomes contained a low amount of diversity with domesticated accessions separated by a maximum of 5 SNPs and little inference on demography could be made. However, in phylogenies the chloroplast genomes separated into two clades, similar to what has been reported for nuclear DNA from *P. miliaceum*. The chloroplast genomes of two wild (undomesticated) accessions of *P. miliaceum* contained a relatively large number of variants, 11 SNPs, not found in the domesticated accessions. These results demonstrate that *P. miliaceum* grains from archaeological sites can preserve DNA for at least 1000 years and serve as a genetic resource to study the domestication of this cereal crop.

## Introduction

Millet is a generic term that refers to a group of small seeded grasses that were important crops in the past and remain a significant source of food and fodder in many areas of the world today^1,2^. The majority of cultivated millets belong to the Paniceae tribe of the Poaceae family and include *Panicum miliaceum* L., *Cenchrus americanus* (L.) R.Br., and *Setaria italica* (L.) P. Beuvois, while *Eleusine coracana* Gaertn. belongs to the Chlorideae clade of Poaceae^3^. Millet, along with other C4 pathway species such as *Zea mays* L., *Saccharum officinarum* L. and *Sorghum bicolor* (L.) Moench, are characterised by their ability to be cultivated in a wide range of environments, requiring modest amounts of water, and having short growing seasons (60–90 days).

Two millets commonly grown throughout much of Eurasia in antiquity were *P. miliaceum* and *S. italica*. Both of these millets were domesticated in Northern China with archaeological evidence indicating that *P. miliaceum* was being farmed as early as 5880 BCE^4–8^, while *S. italica* was first domesticated in the same region at approximately 7000–4000 BCE^9^. From domestication centres in Northern China, the cultivation of *P. miliaceum* and *S. italica* spread to much of Eurasia where these cereals became important food sources^2^. The timing and mechanism by which the cultivation of *P. miliaceum* and *S. italica* moved towards Europe is not well understood but was likely driven by mobile pastoralists who migrated along the mountain foothill ecotone of Central Asia, a path that later became one of the Silk Road trade routes^2,10^.

Areni-1 is a three-chambered karstic cave (also known as Birds’ Cave: 39° 43′ 53″ N, 45° 12′ 13″ E), located on the left bank of the Arpa River basin, a tributary of the river Araxes, within the eastern portion of the modern village of Areni in southern Armenia. Excavations at Areni-1 have uncovered a long history of occupations spanning approximately 6000 years. Plant remains recovered from Areni-1 include vast quantities of well-preserved desiccated and charred seeds, fruits, stones (endocarps), and stems of both wild and cultivated plants^11^. Among the cereals recovered were grains that were identified morphometrically as *P. miliaceum*^11,12^.

The Armenian Highlands, which includes Areni-1, and the Caucasus in general are interesting locations where wild relatives of many cereals are widely represented. The proximity to both the Fertile Crescent a region important for the development of agriculture^13^ and to Silk Road exchange routes^14^ underlies the importance of these locations to processes of domestication, adaptation, and timing of distribution events. Because of this unique relationship of the Armenian Highlands and changes in plant use, the millet grains from Areni-1 provide important information relating to the demographic and evolutionary history of *P. miliaceum*. The excellent preservation of the Areni-1 grains suggested that these samples would contain high levels of DNA and genetic studies of these grains were initiated using the chloroplast genome as the marker^15^.

Chloroplasts are semi-autonomous organelles that provide plants with energy through photosynthesis and contain an independent circular genome. Chloroplast genomes are typically present as double stranded DNA in the range of 107–218 kb that includes two inverted repeat (IR) regions that separate a large and a small single-copy regions^16^. It is widely accepted that the chloroplast evolved from a photosynthetic prokaryote that assimilated with a eukaryotic host more than a billion years ago^17,18^. Owing to various characteristics, chloroplast genes and genomes are extensively used in studies of species identification, evolutionary analyses, and phylogenetics. These attributes include primarily maternal inheritance and thus nonrecombinant^19^, a moderate mutation rate that lies between the mitochondrial and nuclear genomes^20^, and a conserved gene structure and content^21,22^. A particular benefit for ancient DNA (aDNA) studies, is that chloroplast genomes are present as multiple copies in cells which increases the likelihood that chloroplast DNA (cpDNA) will survive in archaeobotanical material.

Using ancient aDNA extraction techniques, hybridization capture enrichment^23^, and high throughput sequencing, we generated three chloroplast genomes from five Areni-1 grains assayed. First, the Areni-1 chloroplast genomes were compared to 11 members of the Paniceae tribe of grasses to verify the morphological identification of the Areni-1 grains and to validate our laboratory and analytical methods. Next, the Areni-1 chloroplasts were compared to similar genomes from 50 modern accessions of *P. miliaceum*. These comparisons to modern accessions sought to provided information on the route cultivated *P. miliaceum* travelled from domestication centres is Northern China eastward and to determine the extent to which the Areni-1 *P. miliaceum* had been taken through the domestication process.

## Results

### Areni-1 millet

**Figure 1:**
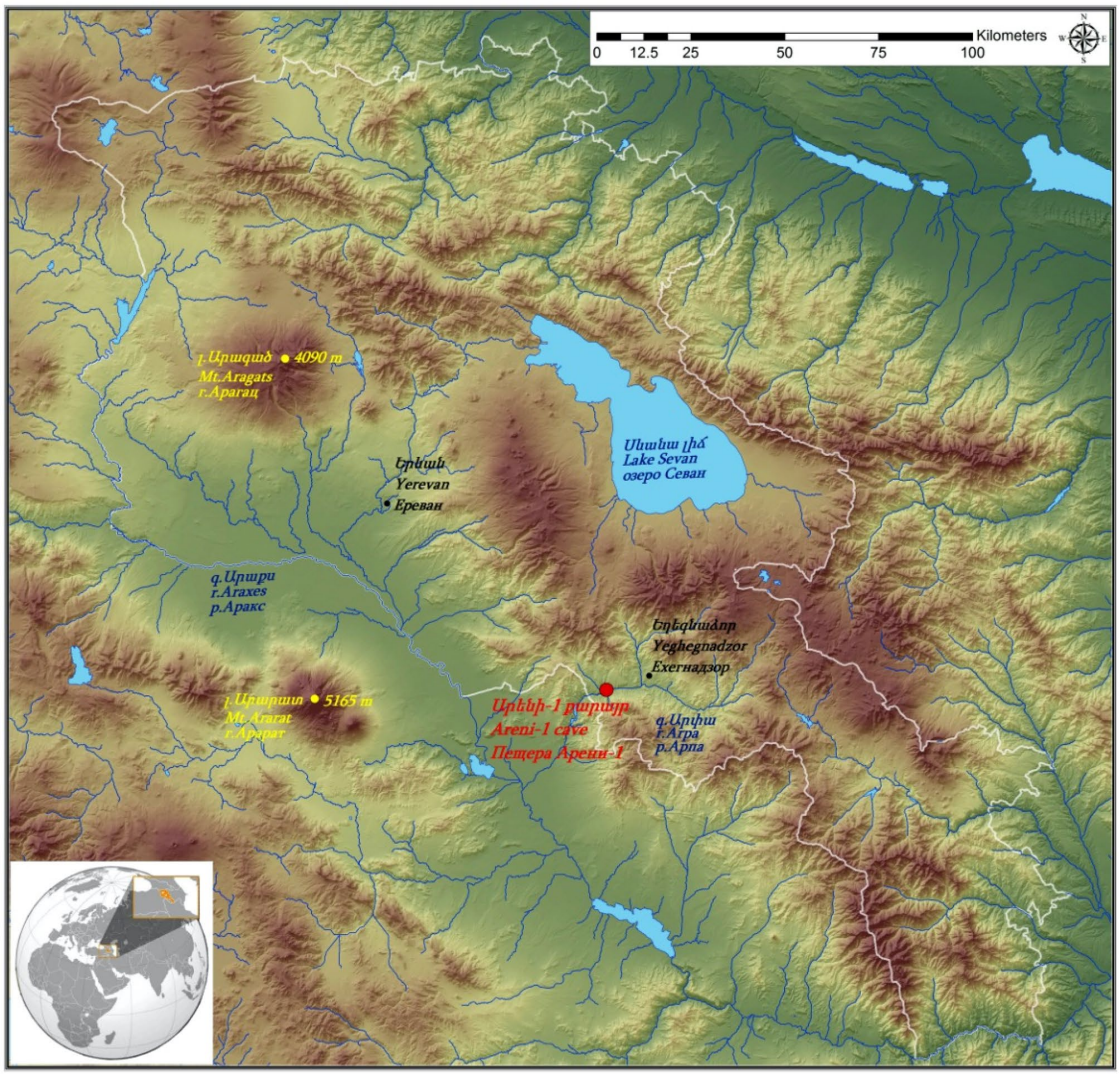
Location of the Areni-1 cave. Small insert at bottom of figure shows location of Armenia within Eurasia. In the large insert, Armenia is shown with the white outline and the location of Areni-1 is indicated in red.

**Figure 2:**
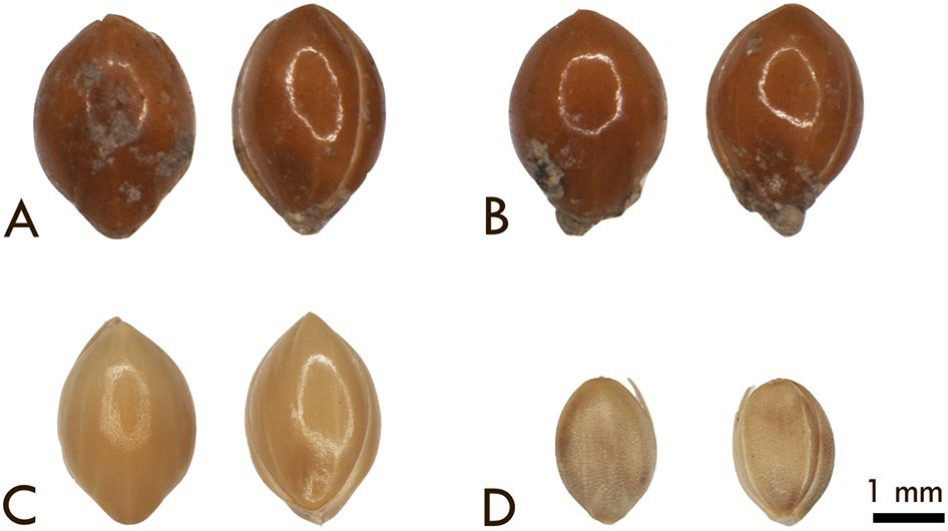
Areni-1 and modern millet grains. Paired photographs of 4 different grains in dorsal (left) and ventral (right) views depicting (**A** and **B**) representative archaeological *Panicum miliaceum* florets from Areni-1; (**C**) modern comparative *P. miliaceum subsp. miliaceum* floret (USDA PI 170588, Turkey); and (**D**) modern comparative *Setaria italica subsp. italica* floret (USDA PI 173103, Turkey). NPGS: U.S. National Plant Germplasm System (https://www.ars-grin.gov/npgs/index.html)

The Areni-1 cave located in southern Armenia produced a vast array botanical material from occupations dating to 4000 BCE (Fig. 1 and Table 1). Exceptionally well preserved, intact *Panicum miliaceum* L. florets were identified from both Trench 1 and 3 based on their morphology (Table 2 and Fig. S1; methods described more fully below). Florets are the basic threshing unit of *P. miliaceum,* exhibiting an indurate, smooth, and shiny palea and lemma tightly enclosing an inner caryopsis, with a pointed apex and relatively blunt proximal end (Fig. 2). The vast majority of ancient millet remains across southwest Asia tend to have been preserved via charring, which frequently damages the palea and lemma, leaving only the caryopsis/seed^24^; the desiccated remains from Areni-1 differ in that the colour and husk (palea and lemma) were maintained (Fig. 2). The lack of charring undoubtedly enhanced the preservation of DNA. The *P. miliaceum* florets from Areni-1 measure roughly 3.3 × 2.2 mm. These dimensions are slightly larger than charred caryopses reported from Mongolia, Afghanistan, Turkey, Iran, and elsewhere^25–27^, but this not surprising given that the medieval remains from Areni-1 represent a long history of selection post-dating earlier domestication events and were preserved via desiccation with the palea and lemma of the floret intact.

**Table 1:** Relevant periods and dates associated with the occupations of the Aren-1 cave complex.

Ages/periods	Inner division	Chronologies, ca.
**Chalcolithic**	Early/Middle	5200–4300 BCE
	Late	4300–3500 BCE
**Bronze**	Early	3500–2400 BCE
	Middle	2400–1500 BCE
	Late	1500–1200 BCE
**Iron**	Early	1200–900 BCE
	Middle	900–700 BCE
	Late	700–600 BCE
Classical	Early	600–200 BCE
	Middle	200–1 BCE
	Late	1–450 CE
**Medieval**	Early	450–900 CE
	High	900–1400 CE
	Late	1400–1700 CE

Periods with occupations at the Areni-1cave are given in bold.

**Table 2:** Archaeological context and associated radiocarbon dates of broomcorn millet grains.

Sample	Location in Areni-1 Cave	Dating laboratory number	Material dated	Date	Calibrated date 1 sigma (68.2%)	Calibrated date 2 sigma (95.4%)
11294a 11294b	Trench 1, Unit 3, Locus 50 (10.01.2009)	AA-107539 X30053	*Vitis* sp. pedicel	873 ± 36 BP	1054–1218 CE	1042–1248 CE
11292a 11293a 11295a	Trench 3, Sq O29/O30 Locus 2, Spit 5	AA-104139 X27645	*Panicum miliaceum* (pool of 3 grains)	1118 ± 35 BP	890–980 CE	780–1020 CE

AMS^14^C determinations for the millet from Areni-1were calibrated using OxCal v. 3.5 based on the last atmospheric dataset OxCal v.3.10^85^ and IntCal13^86^.

To confirm the contextual dating of the Areni-1 *P. miliaceum*,^14^C dating was performed on botanical material associated with the grains. The size of the Arerni-1 *P. miliaceum* did not provide sufficient mass to perform^14^C dating and extraction of aDNA from a single seed, so^14^C dating needed to be conducted indirectly using associated botanical material. The^14^C dating determined the Areni-1 grains were ≈ 1000-years-old and belonged to the Early and High medieval periods (Tables 1 and 2).

### Mapping Areni-1 shotgun data to chloroplast reference genome

In the shotgun libraries, 11292a and 11295a were the only two grains that yielded a significant level of endogenous DNA and produced a large number of unique reads mapping to the chloroplast reference (> 90,000). The remaining shotgun libraries produced more than a thousand-fold fewer unique chloroplast mapped reads (Table 3). The extraction blanks and certain grains (11293a and 11294b) contained a comparatively small number of unique mapped reads in both the shotgun and cpDNA enriched libraries, suggesting that contamination from laboratory sources or between libraries during the processing of the Areni-1 samples was not a substantial issue. In the shotgun libraries, grains 11293a and 11294b both produced fewer mapped reads than the extraction blanks, but these results are likely very stochastic as these libraries contained a very small fraction of mappable reads.

**Table 3:** Mapping statistics of shotgun and cpDNA enriched libraries.

Sample	# Collapsed reads	# Mapped reads	# Unique mapped reads	% Reference covered by least 1 read (sans IRs)
**Shotgun libraries**				
19885 (extraction blank)	6702745	22	14	
11292a	31367680	155496	92560	
11293a	150049	2	2	
11294b	6404935	0	0	
18335 (extraction blank)	6493567	15	9	
11294a	4635555	72	72	
18324 (extraction blank)	6771883	7	7	
11295a	42796618	444954	137838	
**cpDNA enriched libraries**				
19885 (extraction blank)	9896218	1451964	67	
11292a	36092254	21953919	163494	99.77
11293a	35345608	88165	232	
11294b	32245568	269895	1532	
18335 (extraction blank)	77604223	270461	36	
11294a	26076965	7472857	19420	91.28
18324 (extraction blank)	42353298	128226	32	
11295a	41491510	19512189	171867	99.95

Mapping statistics of libraries made from Areni-1 millet grains and mapped to the *Panicum miliaceum* chloroplast reference genome (Genbank#: KU343177.1). Inverted repeat regions (IRs) are two nearly identical loci in the chloroplast genome where the short fragmented aDNA sequencing data could not accurately map (Fig. 2) and were excluded from any analysis. The IRs loci excluded represent bp: 81851 to 104489 and 117101 to 139826 of the reference, which is 139,826 bp in length.

### Mapping of Areni-1 enriched cpDNA data to chloroplast reference genome

**Figure 3:**
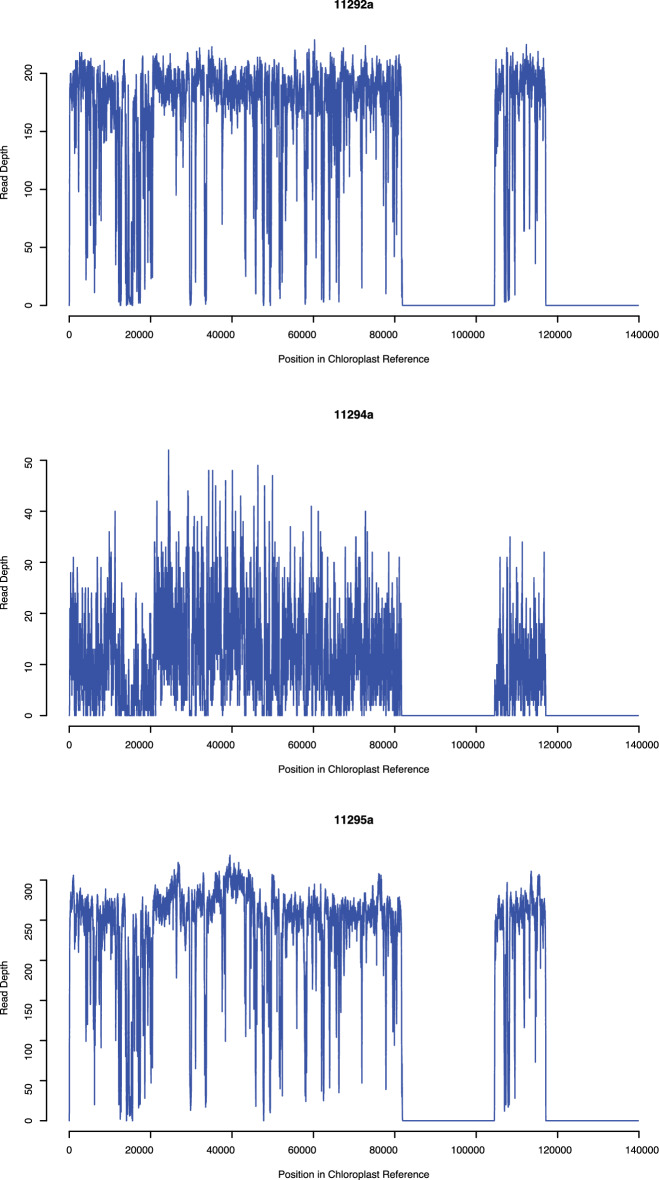
Read depth of mapped cpDNA. Read depth of cpDNA enriched libraries from the three Areni-1 millet grains mapped to the chloroplast reference genome: Genbank# KU343177.1. The IRs are shown in the large tracks of the graph which lack mapped reads.

All ancient shotgun libraries were taken through two rounds of cpDNA hybridization capture regardless of endogenous DNA content in order to treat all samples in a similar manner and maximize the recovery of target sequences. Of the Areni-1 *P. miliaceum* enriched for cpDNA, only three grains (11292a, 11294a, and 11295a) produced libraries that contained a sufficient number of reads that mapped to the chloroplast reference genome (> 19,000) that allowed reconstruction of the Areni-1 chloroplast genomes (Table 3). No reads from the ancient libraries could be mapped to the IRs of the chloroplast genome, because the short-fragmented sequences in this data could not be accurately placed in these nearly identical loci (Fig. 3). As the IRs do not contain any mapped reads, these loci were removed from all further analysis. Libraries 11292a and 11295a covered > 99.7% of the reference (sans IRs) with at least one read, whilst the fraction of the reference covered by at least one read in library 11294a was approximately 8% less than the other two libraries.

### Read length distribution and mapDamage analysis of enriched cpDNA libraries

**Figure 4:**
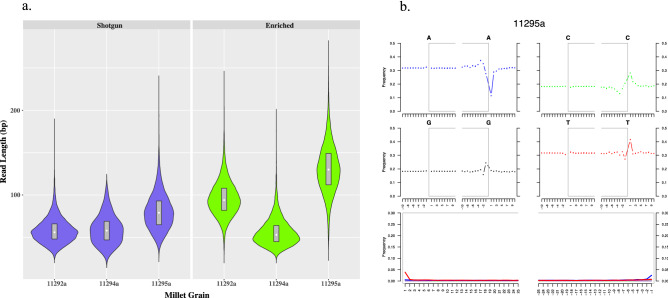
Read length distribution and mapDamage analysis. (**a**) Read length distributions of the shotgun and cpDNA enriched libraries made from three Areni-1 millet grains. All libraries were mapped to the *Panicum miliaceum* chloroplast reference genome: Genbank# KU343177.1. The shape of the violin displays the frequency distribution of the read lengths in each library. Inside each violin, the grey rectangle represents the interquartile range of the read lengths, while the white circle is the median^87^. (**b**) The nucleotide damage pattern of the cpDNA enriched library from the 11295a grain was assayed using mapDamage 2^31^. The enriched library exhibited the C→T nucleotide misincorporations pattern typical of authentic aDNA. The cpDNA libraries from grains 11292a and 112924a gave similar results and are presented in Fig. S2.

To support the ≈1000-year-old^14^C dating of the Areni-1 grains, sequences from the 11292a, 11294a, and 11295a cpDNA enriched libraries were examined for characteristics of authentic aDNA: short read lengths and the presence of deamination miscoding lesions^28,29^. All libraries from the three grains contained reads that were fragmented to a short length, generally < 150 bp (Fig. 4a). Grain 11294a produced the shortest reads, suggesting that this sample was the most degraded of the three. In the cpDNA enriched libraries, the read length of grains 11292a and 11295a was shifted towards longer sequences, which is expected as larger DNA fragments form more stable complexes with enrichment probes and are thus recovered more efficiently in hybridization capture procedures^23^. In contrast, the enriched library for 11294a experienced a downward shift in read length, which likely stems from the stochastic effect of having only 72 unique mapped reads in the shotgun library for this sample. In mapDamage analysis of the cpDNA enriched libraries, the ‘5 ends of reads exhibited the increased levels of C→T misincorporations caused by deamination of cytosine typical of aDNA (Fig. 4b and Fig. S2)^30,31^. MapDamage results are not presented for shotgun data because the library from grain 11294a contained an insufficient number of reads for this analysis. Since direct^14^C dating of the Areni-1 broomcorn millet used for sequencing was not possible due to the small mass of the grains, the fragmentation patterns and miscoding profiles are important data that support the medieval dating of the samples.

### Mapping statistics for Areni-1 enriched cpDNA data

In the mapping of cpDNA enriched libraries, grains 11292a and 11295a produced similar numbers of unique reads (≈ 170,000) that mapped to the chloroplast genome reference, while sample 11294a generated approximately a tenth of the mapped reads observed in the other two grains. The maximum read depth was greater with grains 11292a and 11295a at 229 and 331 reads respectively, compared to 52 reads for 11294a (Fig. 3). Despite the differences in coverage of the chloroplast reference genome and read depth, all three samples produced chloroplast data that allowed for phylogenetic and haplotype network analysis.

### Generation of chloroplast genomes from 50 modern accessions of broomcorn millet

For comparative purposes, chloroplast genomes were generated from 50 accessions of modern *P. miliaceum* (Table S1). These accessions had a mainly Eurasian distribution and were weighted towards Chinese accessions with 29 of the samples originating from various locations within China. Included in these accessions were two wild (undomesticated) broomcorn millet (SampleID 31 and 167) and two accessions that have previously been used to produce nuclear reference genomes (SampleID 69 and T296)^32,33^. The sequencing of these modern accessions generated average read depths of ≥ 1356 reads across the reference chloroplast genome.

### Paniceae phylogenetic analysis

**Figure 5:**
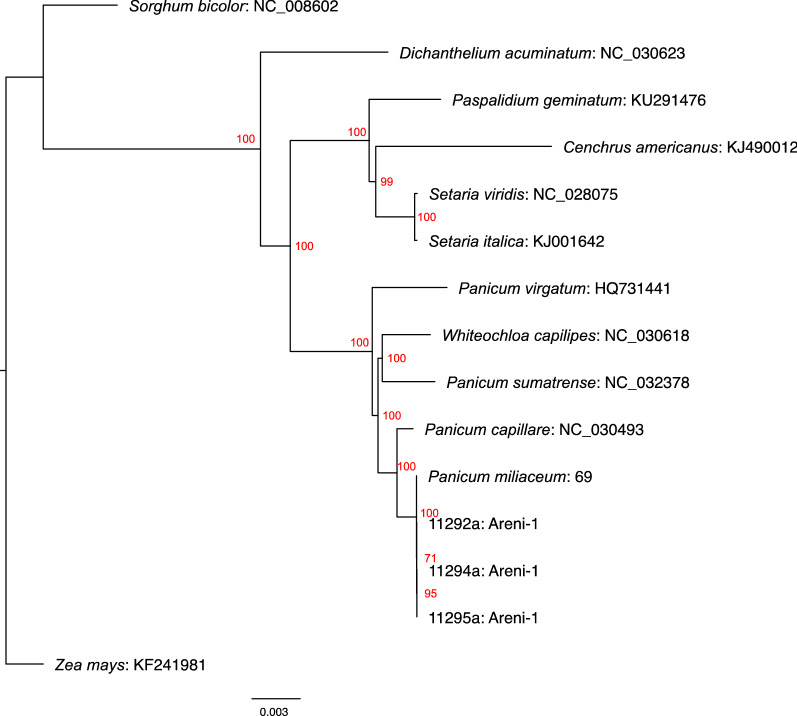
Paniceae chloroplast phylogeny. A phylogeny generated with chloroplast genomes (sans inverted repeat regions) from 11 modern Paniceae species and 3 Areni-1 millet grains using the RAxML program with the GTRGAMMA model^81^. The tree was rooted on the outgroup *Zea mays* (maize). The scale bar at the bottom of the phylogeny represents the substitutions per site.

To confirm the visual identification and the taxonomic placement of the Areni-1 *P. miliaceum* and validate our laboratory and analytical methods, the chloroplast genomes from these medieval grains were compared to similar sequences from 11 members of the Paniceae tribe, including a modern *P. miliaceum* (Table S2). A phylogeny was generated of the Paniceae and Areni-1 chloroplast genomes with maximum likelihood method (Fig. 5). In the tree, the Areni-1 samples and broomcorn millet were placed together on a branch with a bootstrap support of 100, which indicated that the medieval grains were closely related to broomcorn millet and confirmed the taxonomic placement and visual identification of the medieval grains*.* The placement of the *Panicum* and *Setaria* species in the tree suggested the topography produced in this analysis was correct. All the *Panicum* spp. were placed on the same branch within the tree and domesticated *S. italica* formed a clade with the wild antecedent of this cereal, *Setaria viridis*^9^.

### *Panicum miliaceum* phylogenetic and haplotype network analysis

**Figure 6:**
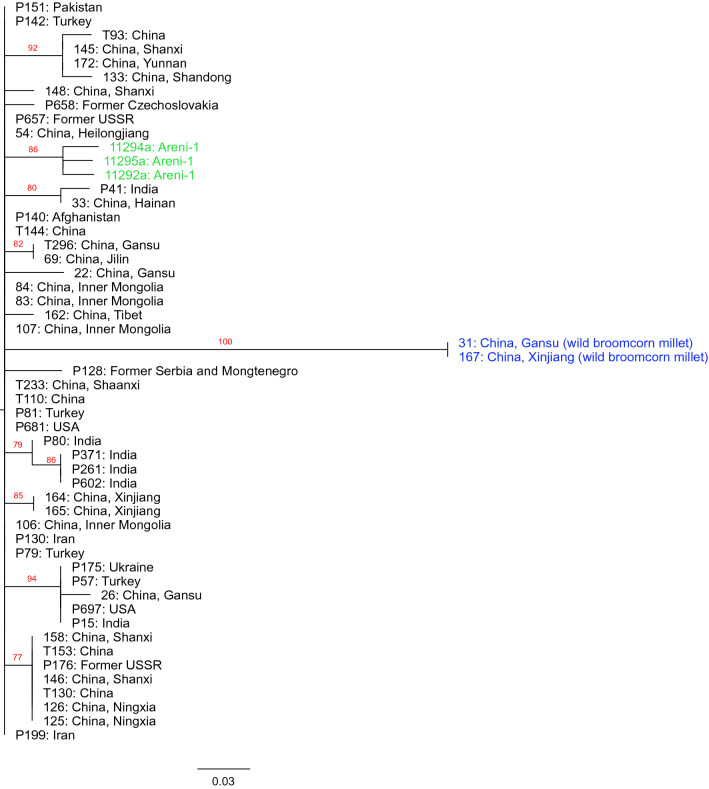
*Panicum miliaceum* maximum likelihood phylogeny. A RaxML maximum likelihood phylogeny generated using the chloroplasts genomes from the three Areni-1 grains and 50 modern accessions of *P. miliaceum* with rooting on the branch containing the two wild accession (31,167). The scale bar at the bottom of the phylogenies represents the substitutions per site. Accessions highlighted in green are the Areni-1 millet and the accession in blue are the wild *P. miliaceum*. Additional Bayesian and neighbour joining phylogenies of this data are given in Fig S3.

At the time the current study was started little was known about *P. miliaceum* chloroplast diversity or what demographic or evolutionary information the chloroplast genome from this cereal could contained. To address these deficiencies, we compared the Areni-1 chloroplast genomes to similar organelle sequences from 50 modern varieties of *P. miliaceum* including two wild accessions. Between the modern accessions and the Areni-1 grains a total of 63 SNPs were identified in the chloroplast genomes and these genotypes were used to generate phylogenies using maximum likelihood, Bayesian, and neighbor joining methods (Fig. 6 and Fig. S3). Generally, across the trees there was low bootstrap support and no geographic structure, so no inference on *P. miliaceum* demographics could be made. However, in the trees produced with the maximum likelihood and Bayesian methodologies, the domesticated accessions were split into two large clades and in each of these phylogenies the Areni-1 chloroplast genomes grouped together as a clade.

**Figure 7:**
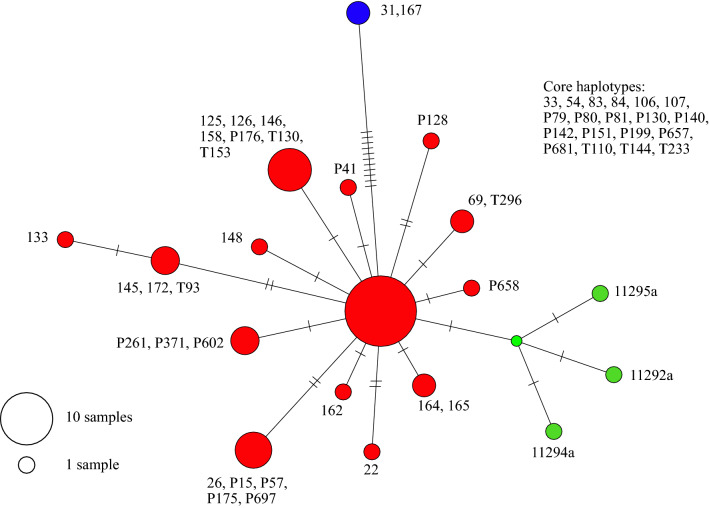
*Panicum miliaceum* haplotype network. A chloroplast genome haplotype network from the three medieval Areni-1 grains and 50 modern accessions of *P. miliaceum* was generated using the PopArt program (http://popart.otago.ac.nz/index.shtml) with the median joining network algorithm. The hash marks on the lines connecting the circles indicate the number of mutations separating the haplotypes. The accessions contained in the core haplotype, the large node at centre of network, are listed in the upper right of the figure. Modern domesticated accessions are given in red, wild modern accessions are given in blue, and the Areni-1 millet are given in green.

The relationship between the modern and Areni-1chloroplast genomes was further investigated in a haplotype network using the PopArt software with the median spanning network algorithm (Fig. 7). In this network, all the domesticated accessions, including the Areni-1 samples, were separated by only 1–5 SNPs. A core haplogroup, represented by the large node at the centre of the network, contained 19 accessions with a wide geographic range across Eurasia. The Areni-1 chloroplast genomes were separated from the core haplogroup by two mutations, one of which was common to all three medieval samples. The largest number of variations was observed in the wild *P. miliaceum*, which were separated from the domesticated accessions by 11–14 SNPs.

## Discussion

In this study, DNA extraction and hybridization capture enrichment techniques were used to recover aDNA from medieval broomcorn millet grains found in Areni-1, a cave site located in the Armenian Highlands a region that has an important history in the domestication and dispersal of many crops. Despite variable quality of aDNA found in the Areni-1 grains, we were able to recover three chloroplast genomes from five grains assayed.

Despite a similar well-preserved physical appearance, the endogenous DNA content varied among the Areni-1 grains used in this study as did the quality of the aDNA. For example, in the millet enriched for cpDNA, grain 11295a contained the highest level of endogenous DNA and the longest read length, while grain 11294a contained the least amount of endogenous DNA with the shortest read length (Table 2, Fig. 3a). The environment of the cave appears to be suited to prevent decomposition of biological material and macromolecules as excavations at Areni-1 have unearthed vertebrate soft tissues including a mummified goat^34^ and brain tissue in a human skull^35^, demonstrating that the conditions in the cave can preserve ephemeral biological material. It is not clear why the Areni-1 millet exhibited different levels of DNA preservation. Grains with poor preservation may have undergone different harvesting, threshing, or other anthropogenic treatments that influenced DNA stability. Post excavation factors, such as storage or handling conditions, may have also influenced the different levels of DNA preservation. Further, the micro-environment has been shown to impact the survival of DNA in mammalian bones^36^ and similar processes may have influenced the decay of aDNA in the Areni-1 millet grains. Local variations in pH, ion concentration, moisture, or other abiotic factors could have effected DNA stability in the Areni-1 millet^37^. Variable preservation of aDNA from grains is not unique to the Areni-1 millet as a study of 6000 year old barley found large differences in recoverable endogenous DNA in grains excavated from the same occupation layer of a cave^38^. Regardless of the varying integrity of the aDNA, the recovery of chloroplast genomes from the Areni-1 samples demonstrates that broomcorn millet grains can, under the right conditions, preserve DNA for at least 1000 years. Even with a poorly preserved sample (11294a), hybridization capture enrichment was able to recover sufficient cpDNA to perform a chloroplast phylogenetic and haplotype analysis.

In the Paniceae grass chloroplast phylogeny (Fig. 5), the Areni-1 and *P. miliaceum* reference clustered with several species native to North America (*Panicum virgatum*, switchgrass; *Panicum capillare*, witchgrass) and *Whiteochloa capillipes*, which is from Australia, New Guinea, and Indonesia. *Panicum sumatrense*, a cereal crop originally from India also clustered with the Areni-1/ *P. miliaceum*. *Panicum* is the largest genera of the grass family Paniceae and has a cosmopolitan distribution so the clustering of the Areni-1/ *P. miliaceum* with widely distributed species is not unusual^39^. While it is unlikely that the specimens from Areni-1 are directly related to these species, it is possible that some of them share a common ancestor from Southeast Asia, further supporting the east to west introduction of *P. miliaceum* in Eurasia. It should be noted that the chloroplast phylogeny indicates a close relationship between broomcorn millet and *P. capillare* (bootstrap support = 100) an observation that supports a previous study of *Panicum* nuclear DNA, which found that diploid *P. capillare* or a close relative is the likely maternal genome donor to tetraploid broomcorn millet^40^.

To investigate the demographic history of *P. miliaceum*, chloroplast phylogenies were generated for the Areni-1 samples and modern accessions using maximum likelihood, Bayesian, neighbour joining methodologies. In these analyses only 63 SNPs were identified in a group that included 48 modern domesticated accessions, two wild accessions, and the three ≈1000-year-old grains from the Areni-1 cave. Accordingly, there was little differences between these chloroplast genomes which was reflected in the phylogenies with low bootstrap support values through much of the trees. No conclusions to be drawn on the demographics of broomcorn millet form these analyses (Fig. 6 and Fig. S3). The inability of chloroplast genomes to resolve intraspecies relationships is not unique to the current study. Research on the genera *Amaranthus* and *Euphrasia* found that whole chloroplast genomes were highly conserved and lacked the power to discriminate between some intraspecies accessions^41,42^. Yet, in all these trees the Areni-1 chloroplast genomes fell within the diversity of domesticated accession, indicating the Areni-1 grains are a fully domesticated variety of *P. miliaceum* and not a wild or partially domesticated form of the cereal.

However, in the maximum likelihood and Bayesian phylogenies, the domesticated accessions were split into two clades with no geographical structure and each clade containing accessions from locations throughout Eurasia (Fig. 6 and Fig. S3a). These results are similar those found in a study by Hunt et al.^43^ which used 16 microsatellite loci across 98 *P. miliaceum* landraces and divided modern broomcorn millet into two clades, one composed solely of Chinese accessions and the other clade contained accessions from throughout Eurasia. It is not clear why the chloroplast phylogenies in the current study lacked a clade of solely Chinese accessions as observed by Hunt et al. 2011. Previous studies have reported incongruent chloroplast and nuclear phylogenies and have attributed these differences to incomplete lineage sorting and introgression/hybridization^44,45^^45^. Similar processes may be involved with the discrepancies between the *P. miliaceum* nuclear and chloroplast genomes, however the low levels of diversity in the chloroplast genomes are likely the main contributing factor for these differences. The *P. miliaceum* chloroplast genomes contained sufficient information for gross scale separation of the different accessions but was insufficient to accurately resolve fine scale relationships.

In the haplotype network (Fig. 7), the majority of the haplotypes were separated by 1–5 SNPs, which did not allow for any conclusions to be drawn about the demographic history of *P. miliaceum*. Wild accessions contained a relatively high number of variants, 11 SNPs, that were not present in the domesticated accessions. Several scenarios that are not mutually exclusive could explain this observation. One possibility is that the wild accessions sampled may not be directly related to the broomcorn lineages that were domesticated. Another possible scenario is the SNPs observed in the wild accessions may represent diversity lost in the chloroplast genome during a domestication bottle neck. Such a loss of diversity has been reported in a study by Leigh et al.^46^, which described a reduction in both alleles and haplotypes in the chloroplast genomes of different wheat species after domestication. However, a much larger sampling of chloroplast genomes from wild accessions will be needed to resolve the relationship of undomesticated and domesticated *P. miliaceum.*

Domestication of crops involve the selection of plant traits that better meet the needs of humans. Chloroplasts are not only responsible for photosynthetic energy production, but are also involved with other cellular functions including synthesis of amino acids, nucleotides, fatty acids, phytohormones, vitamins, and the production of metabolites that are important for plant interaction with biotic and abiotic factors in the environment^16^. Since the chloroplast regulates such a broad range of cellular functions that are essential to *P. miliaceum* for survival and success as a cereal, traits under the control of this organelle will come under selection by humans. The similarity of the Areni-1 chloroplast genomes to those from modern domestications may represent a limited range of genotypes that human find beneficial to *P. miliaceum*.

The chloroplast inheritance pattern will aid in maintaining chloroplast genotypes that are beneficial as a crop. Although unproven in *P. miliaceum*, as far as the authors can determine, maternal inheritance of chloroplast in *P. miliaceum* is the mostly likely scenario because maternal inheritance occurs in most higher plants and maternal inheritance has been shown to be the primary inheritance pattern in *Panicum virgatum*, a relative of *P. miliaceum*^47,48^. With maternal inheritance there is no meiosis or recombination so chloroplast genotypes beneficial to humans will not be broken up and can be more readily maintained in a cultivated plant^49^.

It is possible that mapping of promiscuous DNA that has broken off the chloroplast genome and become incorporated in either the nuclear or mitochondrial genome has influenced the Areni-1 chloroplast results^50,51^. Effort was made to minimize the impact of promiscuous cpDNA on the Areni-1 results by requiring a relatively high read depth (≥ 10) and at least 95% of the reads to call a mutation as an alternative allele. However, as it will be difficult to distinguish between promiscuous cpDNA and DNA from the chloroplast, proving that promiscuous cpDNA has not influenced the current results is extremely challenging. Regardless, the agreement of the morphological identification of the Areni-1 millet as broomcorn millet and the placement of the Areni-1 millet with *P. miliaceum* in the Paniceae chloroplast phylogeny (Fig. 5) indicate that promiscuous cpDNA did not have a significant impact on the interspecies analysis.

In conclusion, here we generated 53 *P. miliaceum* chloroplast genomes, which includes three that are ≈1000-yeaers-old that can be used as resources to study this cereal crop and other C4 plants. A phylogeny generated from these chloroplast genomes divided the domesticated forms of broomcorn millet into two clades, an observation that parallels a report using nuclear DNA. The chloroplast genomes from domesticated accessions displayed low diversity and wild accessions sampled in this study contained SNPs that were outside the diversity of the domesticated forms of *P. miliaceum*. Lastly, this study demonstrates that broomcorn millet grains can preserve significant levels of DNA for hundreds if not thousands of years and can serve as an important genetic resource to investigate the evolution and domestication of this cereal.

## Materials and methods

### Areni-1 archaeology of botanical material

To identify the plant material at Areni-1, a comprehensive archaeobotanical sampling strategy was adopted for the cave sediment. Sediment samples with a volume of 5 L were collected throughout the excavation and separated using dry sieving instead of flotation. The majority of plant remains found at Areni-1 were desiccated and contact with water was likely to damage this material. After passing each sediment sample through a 1 mm sieve, material > 1 mm was kept for study and < 1 mm was discarded, which allowed for some small weed seeds and plant parts to be lost. Similar approaches to sieving have been used at other sites that contained copious amounts of desiccated plant material^52^. Sieved material was hand sorted into categories of plant remains, bones, and pottery fragments. Millet grains were among the plant material recovered from Areni-1 excavations, which also included other domesticated cereals including wheat and barley. To date, all millet found at Areni-1 came from Trench 1 and 3 within the cave site and in stratigraphic horizons attributed to medieval occupations^11^.

### Areni-1 millet grain morphology and identification

Identification of the Areni-1 millet grains were made by comparing morphological features of the ancient florets with modern *Panicum, Echinochloa,* and *Setaria* specimens accessioned from Turkey and Armenia (curated within the Archaeobotany reference collection at the University of Connecticut). To help further hone the identification, a number of images, keys, and textual descriptions of morphological features and species distributions were also consulted, including reference manuals^27,53–56^, the *Flora of Armenia*^57^, *Flora of Turkey*^58^, *Flora of Iraq*^59^, and archaeobotanical publications detailing various additional Paniceae species found across Asia and Europe^25,60–64^. Textual descriptions within flora typically provide size ranges for spikelets rather than florets or caryopses. These descriptions remained helpful, however, given that the spikelet lengths of many taxa (particularly within the *Setaria* genus), were shorter than the florets observed here, and could easily be eliminated. Length to breadth ratios were also considered along with the surface texture of the florets. The Areni-1 millet grains were photographed and measured using a Nikon AZ microscope with associated NIS Elements software.

### Carbon dating of botanical material from Areni-1

A single *Vitis* sp. pedicel from Trench 1 and a pool of three millet grains from Trench 3 (Fig. S1) were sent to the NSF Arizona AMS Laboratory (University of Arizona) for radiocarbon dating. Fraction Modern Carbon and Radiocarbon Age were calculated as weighted averages of combined machine runs to reduce overall error.

### Areni-1 millet library construction and cpDNA enrichment

For genetic studies, five millet grains were chosen at random for the extraction and enrichment of cpDNA (Table 2). Extraction of aDNA and construction of Illumina libraries from the Areni-1 millet was performed in a dedicated low-DNA laboratory where no work had previously been done with millet. Isolation of aDNA from the Areni-1 grains and negative blank controls was performed with a two-step protocol, which has been previously described^65–67^. Shotgun and cpDNA enriched libraries were generated using standard aDNA methods and a detailed description of these protocols is given in the Supplemental Methods.

### Mapping of Areni-1 millet sequencing data

Raw FASTQ files demultiplexed on the i7 adapter index were obtained directly from the sequencer. Run-specific FASTQ files were demultiplexed by the internal barcodes using Sabre 1.0 (https://github.com/najoshi/sabre). AdapterRemoval v2.2.1^68^ was used to trim adapters, collapse reads, discard reads < 25 bp, and remove reads of low quality (< 4). Collapsed reads were mapped to a *P. miliaceum* chloroplast reference genome (Genbank #: KU343177.1) using BWA aln (v0.5.11-foss-2016b) with parameters recommended for aDNA (-l 1024 -n 0.01 -o 2) and a mapping quality (MAPQ) $\ge$ 30^69,70^. Duplicate reads were removed from the mapped data using Picard Tools (v 2.2.4: https://broadinstitute.github.io/picard/index.html). Levels of nucleotide misincorporation caused by deaminated cytosine were assayed using mapDamage 2.0 in cpDNA enriched libraries^31^. Read depths were extracted from cpDNA enriched mapped data using SAMTools (v1.3.1-foss-2016a)^71^ and plotted using R (v3.4.2)^72^. Read length distributions for the shotgun and cpDNA enriched libraries were produced using SeqKit (v 0.7.2)^73^ and SAMTools and plotted with R using the ggplot2 package. For all libraries, except the shotgun library for 11294a, read length analysis was performed using 19,000 reads, a number based on the smallest number of mapped reads produced by a library in this dataset. The shotgun library for 11294a produced 72 mapped reads and this number was used for the read length analysis.

### Sequencing of modern broomcorn millet accessions

DNA was extracted from seedling leaves of 50 modern broomcorn millet accessions (Table S1) using the DNAeasy Plant Mini Kit (QIAGEN) and the provided instructions. Shotgun libraries were constructed from the isolated DNA using a NEBNext Ultra II FS DNA Library Prep Kit for Illumina (New England Biolabs) using the protocol provided by the manufacture. Sequencing of the modern libraries was performed at Core Facility for Genomics at Shanghai Center for Plant Stress Biology (Shanghai, China) on an Illumina HiSeq 2500 platform using 2 × 125 bp (250 cycle) chemistry.

### Mapping of modern broomcorn millet sequencing data

Fastq reads from the modern broomcorn millet accessions were firstly mapped to a broomcorn millet nuclear reference genome (NCBI Assembly: GCA_003046395.2) using BWA mem (v0.5.11-foss-2016b: https://arxiv.org/abs/1303.3997v2). From the resulting bam file, reads that did not map to the nuclear reference were extracted using SAMTools (v1.12) and remapped to the broomcorn millet chloroplast genome (Accession: KU343177.1) using BWA mem. Duplicates were marked with Picard Tools to prevent processing of these reads in downstream analysis. Mapping to the chloroplast reference genome produced average read depths of between 1356 and 4548 across the chloroplast genome (Table S2).

### Variant calling in the Areni-1 and modern millet sequencing data

Variants were called in parallel in the Areni-1 and modern millet using freebayes (v 1.0.2-GCC-4.9.3-binutils-2.25: Garrison and Marth, 2012, arXiv:1207.3907) with the following parameters: --min-base-quality 30 --min-coverage 10 --hwe-priors-off --ploidy 1 --pooled-continuous --genotype-qualities --min-alternate-fraction 0.95 to generate vcf files. The value for the --min-alternate-fraction was chosen to mask polymorphic sites caused by promiscuous cpDNA (chloroplast DNA that has migrated to the nuclear or mitochondrial genomes)^74^. Freebayes automatically disregards reads marked as duplicate, so only unique reads from the modern millet were analysed. Chloroplast genome consensus sequences were generated for the Areni-1 millet using the FastaAlternateReferenceMaker function of GATK (v 3.5-Java-1.8.0_71)^75^ using the freebayes vcf files and the KU343177.1 reference. Two sections of the Areni-1 consensus sequences, bp 81851 to 104489 and 117101 to 139826, were removed with Geneious Prime (Build 2019-04-26) using 11295a as a guide^76^, because these regions represent the IRs of the chloroplast genome where ancient reads could not be mapped (Fig. 3).

### Paniceae phylogenetic analysis

Chloroplast reference genomes for 10 members of the Paniceae tribe of grasses (which includes members of the *Panicum* genus) were downloaded from the NCBI website (Table S1). The reference sequence for the *Zea mays* chloroplast genome was also downloaded for use as an out-group. Although the Grass Phylogeny Working Group II, which used three genetic markers, placed *Whiteochloa capillipes* in the Cenchrinae subtribe^77^, this species was included in the analysis because a more recent study of complete chloroplast genomes placed *W. capillipes* in the Paniceae subtribe^78^. The Areni-1 consensus sequences and the grass references were aligned using MAFFT (v 7.130-foss-2016b-with-extensions) and the default settings^79^.

*P. miliaceum* variety 69 from the modern accessions (Table S2) was included the MAFFT alignment to serve as the broomcorn millet chloroplast genome. After alignment, the Paniceae genomes were trimmed of the IRs with Geneious Prime using sample 11295a as before. Poorly aligned regions were removed from the alignment using the trimAl program with the default parameters on the Phylemon 2.0 website (http://phylemon2.bioinfo.cipf.es)^80^. A maximum likelihood tree was built for the alignment with RAxML^81^ (v. 8.2.10) with 1,000 rapid bootstraps and the “GTRGAMMA” model. Tree visualization was performed with FigTree (v 1.4.3; http://tree.bio.ed.ac.uk/software/figtree/) with rooting on the branch containing the maize chloroplast genome.

### Broomcorn millet phylogenetic and haplotype analysis

The translated genotypes of the Areni-1 and modern millet were extracted from the freebayes vcf file and exported as a multiple sequence file using bcftools (v1.12)^71^. An MAFFT alignment with the IRs and poorly aligned regions removed was produced as before. Phylogenies were then produced for the chloroplast genomes using three methodologies: (1) maximum likelihood using RAxML as describe above; (2) Bayesian using MrBayes (v3.2.7a, https://nbisweden.github.io/MrBayes/download.html with 5 million generations^82^; (3) neighbor joining MEGA11 (https://megasoftware.net/) with 1000 bootstrap replicates^83^. Trees were generated for the phylogenies using FigTree as above. A PHYLIP format file generated from the MAFFT alignment using Geneious Prime was used to produce a haplotype network in the PopART program (http://popart.otago.ac.nz/index.shtml) with the median joining network algorithm^84^.

### Use of plant material

The collection and use of the medieval and modern millet grains complied with all relevant institutional, national, and international guidelines and legislation on the study of plant material. No permission was needed for any of the millet grains used in this study.

## Supplementary Information

Supplementary Information.

## Data Availability

Sequencing data for the Areni-1 samples can be found at the NCBI website under BioProject: PRJNA575768 and the chloroplast sequencing data for the 50 modern broomcorn millet accessions are deposited at figshare: https://doi.org/10.6084/m9.figshare.14914605.

## References

[1] Habiyaremye C (2017). Proso Millet (*Panicum miliaceum* L.) and Its potential for cultivation in the Pacific Northwest, U.S.: A review. Front. Plant Sci..

[2] Miller NF, Spengler RN, Frachetti M (2016). Millet cultivation across Eurasia: Origins, spread, and the influence of seasonal climate. The Holocene.

[3] Cannarozzi G (2014). Genome and transcriptome sequencing identifies breeding targets in the orphan crop tef (Eragrostis tef). BMC Genomics.

[4] Motuzaite-Matuzeviciute G, Staff RA, Hunt HV, Liu X, Jones MK (2013). The early chronology of broomcorn millet (*Panicum miliaceum*) in Europe. Antiquity.

[5] Lu H (2009). Earliest domestication of common millet (*Panicum miliaceum*) in East Asia extended to 10,000 years ago. Proc. Natl. Acad. Sci..

[6] Hunt HV (2018). Genetic evidence for a western Chinese origin of broomcorn millet (*Panicum miliaceum*). The Holocene.

[7] Leipe C, Long T, Sergusheva EA, Wagner M, Tarasov PE (2019). Discontinuous spread of millet agriculture in eastern Asia and prehistoric population dynamics. Sci. Adv..

[8] Stevens CJ, Shelach-Lavi G, Zhang H, Teng M, Fuller DQ (2020). A model for the domestication of *Panicum miliaceum* (common, proso or broomcorn millet) in China. Veg. Hist. Archaeobotany.

[9] Diao, X. & Jia, G. In *Plant Genetics and Genomics: Crops and Models* Vol. Volume 19 (ed Richard, A. J.) Ch. 4, 61–72 (Springer International Publishing, Berlin, 2017).

[10] Spengler R (2014). Early agriculture and crop transmission among Bronze Age mobile pastoralists of Central Eurasia. Proc. R. Soc. B Biol. Sci..

[11] Smith, A., Bagoyan, T., Gabrielyan, I., Pinhasi, R. & Gasparyan, B. In *Stone Age of Armenia, A Guide-book to the Stone Age Archaeology in the Republic of Armenia* (eds Boris, G. & Makoto, A.) 233–260 (Center for Cultural Resource Studies, Kanazawa University, 2014).

[12] Zohary, D., Hopf, M. & Weiss, E. *Domestication of Plants in the Old World : The Origin and Spread of Domesticated Plants in Southwest Asia, Europe, and the Mediterranean Basin*. Fourth edition edn, (Oxford University Press, Oxford, 2013).

[13] Zeder MA (2008). Domestication and early agriculture in the Mediterranean Basin: Origins, diffusion, and impact. Proc. Natl. Acad. Sci..

[14] Christian D (2000). Silk roads or steppe roads? The silk roads in world history. J. World Hist..

[15] Wang Y (2021). Chloroplast genome variation and phylogenetic relationships of Atractylodes species. BMC Genomics.

[16] Daniell H, Lin C-S, Yu M, Chang W-J (2016). Chloroplast genomes: Diversity, evolution, and applications in genetic engineering. Genome Biol..

[17] Sánchez-Baracaldo P, Raven-John A, Pisani D, Knoll-Andrew H (2017). Early photosynthetic eukaryotes inhabited low-salinity habitats. Proc. Natl. Acad. Sci..

[18] Llorente B (2021). Homecoming: rewinding the reductive evolution of the chloroplast genome for increasing crop yields. Nat. Commun..

[19] Birky CW (1995). Uniparental inheritance of mitochondrial and chloroplast genes: Mechanisms and evolution. Proc. Natl. Acad. Sci. USA.

[20] Smith DR (2015). Mutation rates in plastid genomes: They are lower than you might think. Genome Biol. Evol..

[21] Mower, J. P. & Vickrey, T. L. In *Advances in Botanical Research* Vol. 85 (eds Shu-Miaw, C. & Robert, K. J.) 263–292 (Academic Press, Hoboken, 2018).

[22] Gao L-Z (2019). Evolution of Oryza chloroplast genomes promoted adaptation to diverse ecological habitats. Commun. Biol..

[23] Brotherton P (2013). Neolithic mitochondrial haplogroup H genomes and the genetic origins of Europeans. Nat. Commun..

[24] Filipović D (2020). New AMS 14C dates track the arrival and spread of broomcorn millet cultivation and agricultural change in prehistoric Europe. Sci. Rep..

[25] Korolyuk EA, Krasnikov AA, Polosmak NV (2018). Panicoids in Xiongnu burial ground (Mongolia, First Century AD): Problems of identification. Turczaninowia.

[26] Nesbitt M, Summers GD (1988). Some recent discoveries of millet (*Panicum miliaceum* L. and *Setaria italica* (L.) P. Beauv) at excavations in Turkey and Iran. Anatol. Stud..

[27] Motuzaite-Matuzeviciute G, Hunt HV, Jones MK (2012). Experimental approaches to understanding variation in grain size in *Panicum miliaceum* (broomcorn millet) and its relevance for interpreting archaeobotanical assemblages. Veg. Hist. Archaeobotany.

[28] Dabney J, Meyer M, Pääbo S (2013). Ancient DNA damage. Cold Spring Harb. Perspect. Biol..

[29] Armbrecht L, Hallegraeff G, Bolch CJS, Woodward C, Cooper A (2021). Hybridisation capture allows DNA damage analysis of ancient marine eukaryotes. Sci. Rep..

[30] Brotherton P (2007). Novel high-resolution characterization of ancient DNA reveals C > U-type base modification events as the sole cause of post mortem miscoding lesions. Nucleic Acids Res..

[31] Jónsson H, Ginolhac A, Schubert M, Johnson PLF, Orlando L (2013). mapDamage2.0: Fast approximate Bayesian estimates of ancient DNA damage parameters. Bioinformatics.

[32] Shi J (2019). Chromosome conformation capture resolved near complete genome assembly of broomcorn millet. Nat. Commun..

[33] Zou C (2019). The genome of broomcorn millet. Nat. Commun..

[34] Zarikian N, Gasparyan B (2016). Micromammal remains from Areni-1 Cave, Armenia. IOSR J. Human. Soc. Sci..

[35] Wilkinson KN (2012). Areni-1 Cave, Armenia: A Chalcolithic-Early Bronze Age settlement and ritual site in the southern Caucasus. J. Field Archaeol..

[36] Campana MG, McGovern T, Disotell T (2014). Evidence for differential ancient DNA survival in human and pig bones from the Norse North Atlantic. Int. J. Osteoarchaeol..

[37] Pääbo S (2004). Genetic analyses from ancient DNA. Annu. Rev. Genet..

[38] Mascher, M. *et al.* Genomic analysis of 6,000-year-old cultivated grain illuminates the domestication history of barley. *Nature Genet.* **48**, 1089–1093 10.1038/ng.3611, http://www.nature.com/ng/journal/v48/n9/abs/ng.3611.html#supplementary-information (2016). 10.1038/ng.3611 27428749

[39] Aliscioni SS, Giussani LM, Zuloaga FO, Kellogg EA (2003). A molecular phylogeny of Panicum (Poaceae: Paniceae): Tests of monophyly and phylogenetic placement within the Panicoideae. Am. J. Bot..

[40] Hunt HV (2014). Reticulate evolution in Panicum (Poaceae): The origin of tetraploid broomcorn millet *P. miliaceum*. J. Exp. Botany.

[41] Viljoen E, Odeny DA, Coetzee MPA, Berger DK, Rees DJG (2018). Application of chloroplast phylogenomics to resolve species relationships within the plant genus Amaranthus. J. Mol. Evol..

[42] Zhou T (2019). The complete chloroplast genome of *Euphrasia regelii*, pseudogenization of ndh genes and the phylogenetic relationships within Orobanchaceae. Front. Genet..

[43] Hunt HV (2011). Genetic diversity and phylogeography of broomcorn millet (*Panicum miliaceum* L.) across Eurasia. Mol. Ecol..

[44] Yu W-B, Huang P-H, Li D-Z, Wang H (2013). Incongruence between Nuclear and chloroplast DNA phylogenies in pedicularis section cyathophora (Orobanchaceae). PLoS ONE.

[45] Nge FJ, Biffin E, Thiele KR, Waycott M (2021). Reticulate evolution, ancient chloroplast haplotypes, and rapid radiation of the australian plant genus Adenanthos (Proteaceae). Front. Ecol. Evol..

[46] Leigh FJ (2013). Using diversity of the chloroplast genome to examine evolutionary history of wheat species. Genet. Resour. Crop Evol..

[47] Park H-S (2021). Inheritance of chloroplast and mitochondrial genomes in cucumber revealed by four reciprocal F1 hybrid combinations. Sci. Rep..

[48] Martínez-Reyna JM, Vogel KP, Caha C, Lee DJ (2001). Meiotic stability, chloroplast DNA polymorphisms, and morphological traits of upland × lowland switchgrass reciprocal hybrids. Crop Sci..

[49] Greiner S, Sobanski J, Bock R (2015). Why are most organelle genomes transmitted maternally?. BioEssays News Rev. Mol. Cell. Dev. Biol..

[50] Zhang G-J (2020). Nuclear integrants of organellar DNA contribute to genome structure and evolution in plants. Int. J. Mol. Sci..

[51] Roark LM, Hui AY, Donnelly L, Birchler JA, Newton KJ (2010). Recent and frequent insertions of chloroplast DNA into maize nuclear chromosomes. Cytogenet. Genome Res..

[52] Van der Veen, M. *Consumption, Trade and Innovation. Exploring the Botanical Remains from the Roman and Islamic Ports at Quseir a-Qadim, Egypt*. 20–33 (Africa Magna Verlag, 2011).

[53] Fuller, D. Q. In http://www.homepages.ucl.ac.uk/~tcrndfu/Abot/Millet%20Handout06.pdf (ed University College London. Institute of Archaeology) (2006).

[54] Nesbitt M (2006). Identification Guide for Near Eastern Grass Seeds.

[55] Cappers, R. T. J., Neef, R. & Bekker, R. M. *Digital Atlas of Economic Plants. Volume 2b: Icacinaceae–Zygophyllaceae*. (Barkhuis & Groningen University Library, 2009).

[56] Neef, R., Cappers, R. T. J. & Bekker, R. M. *Digital Atlas of Economic Plants in Archaeology*. (Barkhuis & Groningen University Library, 2012).

[57] Agababian, M. Y. *et al. Flora of Armenia Vol 11. Poaceae [in Russian]*. (A.R.G. Gantner Verlag KG, 2010).

[58] Davis, P. H. (Edinburgh University Press, Edinburgh, 1985).

[59] Bor, N. L. *Flora of Iraq Vol. 9*. (Ministry of Agriculture, Republic of Iraq, 1968).

[60] Nasu H, Momohara A, Yasuda Y, He J (2007). The occurrence and identification of *Setaria italica* (L.) P. Beauv (foxtail millet) grains from the Chengtoushan site (ca 5800 cal B.P.) in central China, with reference to the domestication centre in Asia. Veg. Hist. Archaeobotany.

[61] Tsang C-H (2017). Broomcorn and foxtail millet were cultivated in Taiwan about 5000 years ago. Bot. Stud..

[62] Fukunaga K, Kawase M, Sakamoto S (1997). Variation of caryopsis length and width among landraces of foxtail millet, *Setaria italica* (L.) P. Beauv. Jpn. J. Trop. Agric..

[63] Stevens CJ, Shelach-Lavi G, Zhang H, Teng M, Fuller DQ (2021). A model for the domestication of *Panicum miliaceum* (common, proso or broomcorn millet) in China. Veg. Hist. Archaeobotany.

[64] Jiang H, Zhang Y, Lü E, Wang C (2015). Archaeobotanical evidence of plant utilization in the ancient Turpan of Xinjiang, China: A case study at the Shengjindian cemetery. Veg. Hist. Archaeobotany.

[65] Chomczynski P, Mackey K, Drews R, Wilfinger W (1997). DNAzol®: A reagent for the rapid isolation of genomic DNA. Biotechniques.

[66] Rohland N, Glocke I, Aximu-Petri A, Meyer M (2018). Extraction of highly degraded DNA from ancient bones, teeth and sediments for high-throughput sequencing. Nat. Protoc..

[67] Richards SM (2019). Low-cost cross-taxon enrichment of mitochondrial DNA using in-house synthesised RNA probes. PLoS ONE.

[68] Schubert M, Lindgreen S, Orlando L (2016). AdapterRemoval v2: Rapid adapter trimming, identification, and read merging. BMC. Res. Notes.

[69] Schubert M (2012). Improving ancient DNA read mapping against modern reference genomes. BMC Genomics.

[70] Li H, Durbin R (2009). Fast and accurate short read alignment with Burrows-Wheeler transform. Bioinformatics.

[71] Li H (2009). The sequence alignment/map format and SAMtools. Bioinformatics.

[72] A language and environment for statistical computing (R Foundation for Statistical Computing, Vienna, Austria, 2019).

[73] Shen W, Le S, Li Y, Hu F (2016). SeqKit: A cross-platform and ultrafast toolkit for FASTA/Q file manipulation. PLoS ONE.

[74] Scarcelli N (2016). Intra-individual polymorphism in chloroplasts from NGS data: Where does it come from and how to handle it?. Mol. Ecol. Resour..

[75] McKenna A (2010). The Genome Analysis Toolkit: A MapReduce framework for analyzing next-generation DNA sequencing data. Genome Res..

[76] Kearse M (2012). Geneious Basic: An integrated and extendable desktop software platform for the organization and analysis of sequence data. Bioinform. (Oxf., Engl.).

[77] Edwards EJ (2012). New grass phylogeny resolves deep evolutionary relationships and discovers C4 origins. New Phytol..

[78] Burke SV (2016). Evolutionary relationships in Panicoid grasses based on plastome phylogenomics (Panicoideae; Poaceae). BMC Plant Biol..

[79] Katoh K, Standley DM (2013). MAFFT multiple sequence alignment software version 7: Improvements in performance and usability. Mol. Biol. Evol..

[80] Capella-Gutiérrez S, Silla-Martínez JM, Gabaldón T (2009). trimAl: A tool for automated alignment trimming in large-scale phylogenetic analyses. Bioinform.s (Oxf., Engl.).

[81] Stamatakis A (2014). RAxML version 8: A tool for phylogenetic analysis and post-analysis of large phylogenies. Bioinformatics.

[82] Ronquist F (2012). MrBayes 3.2: Efficient Bayesian phylogenetic inference and model choice across a large model space. Syst. Biol..

[83] Tamura K, Stecher G, Kumar S (2021). MEGA11: Molecular evolutionary genetics analysis version 11. Mol. Biol. Evol..

[84] Kong S, Sánchez-Pacheco SJ, Murphy RW (2016). On the use of median-joining networks in evolutionary biology. Cladistics.

[85] Ramsey CB, Lee S (2013). Recent and planned developments of the program OxCal. Radiocarbon.

[86] Reimer PJ (2013). IntCal13 and Marine13 radiocarbon age calibration curves 0–50,000 years cal BP. Radiocarbon.

[87] Hintze JL, Nelson RD (1998). Violin plots: A box plot-density trace synergism. Am. Stat..

