# Supplementary material for: Recovery of chloroplast genomes from medieval millet grains excavated from the Areni-1 cave in southern Armenia
Source: Sci Rep. 2022 Sep 7;12:15164. doi: 10.1038/s41598-022-17931-4 (PMC9452526; doi:10.1038/s41598-022-17931-4)
Supplement: Supplementary file 1 — Supplementary Information. [file 41598_2022_17931_MOESM1_ESM.docx]

Supplementary Information

Recovery of chloroplast genomes from medieval millet grains excavated from the Areni-1 cave in southern Armenia

Stephen M. Richards^1†^, Leiting Li^2†^, James Breen^1*^, Nelli Hovhannisyan^3^, Oscar Estrada^1**^, Boris Gasparyan^4^, Matthew Gilliham^5^, Alexia Smith^6^, Alan Cooper^7,8‡^, and Heng Zhang^2‡^

1 School of Biological Science, The University of Adelaide, Adelaide, Australia

2 National Key Laboratory of Plant Molecular Genetics, Shanghai Center for Plant Stress Biology, CAS Center for Excellence in Molecular Plant Sciences, Chinese Academy of Sciences, Shanghai, China

3 Yerevan State University, Yerevan, Armenia

4 National Academy of Sciences of the Republic of Armenia, Institute of Archaeology and Ethnography, Yerevan, Armenia

5 ARC Centre of Excellence in Plant Energy Biology, Waite Research Institute & School of Agriculture, Food, and Wine, The University of Adelaide, Waite Campus, Glen Osmond, Australia

6 University of Connecticut, Department of Anthropology, Connecticut, USA

7 BlueSky Genetics, Ashton, South Australia, Australia

8 South Australian Museum, Adelaide, South Australia, Australia

^†^Contributed equally to this manuscript

^‡^ Contributed equally to this manuscript

* Current affiliation: Telethon Kids Institute, Australian National University

** Current affiliation: Grupo de Agrobiotecnología, Instituto de Biología, Universidad de Antioquia, Medellín, Colombia

Corresponding authors:

Stephen M. Richards ([smr3434@gmail.com](mailto:smr3434@gmail.com)); Heng Zhang (hengzhang@psc.ac.cn)

**Figure S1. Schematic of the Areni-1 cave and locations where millet grains were excavated**

**a.** Schematic map of the Areni-1 cave showing the various excavation trenches in red; **b**. Trench 1 where millet grains 11294a and 11294b were excavated; **c.** rounded medieval structure in Trench 3 were grains 11292a, 11293a, and 11295a were excavated. Figure S1a created with ArcGIS version 10.4 (https://www.esri.com). © Boris Gasparyan.

**Figure S2. Additional mapDamage plots**

The nucleotide damage patterns of the cpDNA enriched libraries from the Areni-1 grains 11292a and 11294 were generated using the mapDamage 2 program ^36^. These enriched libraries exhibited the C → T nucleotide misincorporations pattern typical of authentic aDNA.

**a)**

**b)**

**Figure S3. Additional *Panicum miliaceum* phylogenies**

Phylogenies of the the chloroplasts genomes from the three Areni-1 grains and 50 modern accessions of *P. miliaceum* with rooting on the branch containing the two wild accession (31,167). The scale bar at the bottom of the phylogenies represents the substitutions per site. Accessions highlighted in green are the Areni-1 millet and the accession in blue are the wild *P. miliaceum.* a) Bayesian generated with MrBayes; b) neighbour joining generated with MEGA11

Table S1. Modern accession of broomcorn millet

| SampleID | Source (accession ID) | Collected from | Average read depth over chloroplast genome |
| --- | --- | --- | --- |
| 22 | CGRIS (00002878) | China, Gansu | 2781 |
| 26 | CGRIS (00002997) | China, Gansu | 2657 |
| 31 | CGRIS (00006767): wild broomcorn millet | China, Gansu | 4548 |
| 33 | CGRIS (00006650) | China, Hainan | 3175 |
| 54 | CGRIS (00000183) | China, Heilongjiang | 4497 |
| 69 | CGRIS (00000390) ^1^ | China, Jilin | 1975 |
| 83 | CGRIS (00000478) | China, Inner Mongolia | 3428 |
| 84 | CGRIS (00000482) | China, Inner Mongolia | 4196 |
| 106 | CGRIS (00002340) | China, Inner Mongolia | 3351 |
| 107 | CGRIS (00002368) | China, Inner Mongolia | 4152 |
| 125 | CGRIS (00002651) | China, Ningxia | 3054 |
| 126 | CGRIS (00006776) | China, Ningxia | 3479 |
| 133 | CGRIS (00004326) | China, Shandong | 2051 |
| 145 | CGRIS (00001002) | China, Shanxi | 3336 |
| 146 | CGRIS (00001145) | China, Shanxi | 3028 |
| 148 | CGRIS (00003209) | China, Shanxi | 2168 |
| 158 | CGRIS (00003803) | China, Shanxi | 2564 |
| 162 | CGRIS (00006681) | China, Tibet | 2294 |
| 164 | CGRIS (00003082) | China, Xinjiang | 3652 |
| 165 | CGRIS (00003124) | China, Xinjiang | 3787 |
| 167 | CGRIS (00006766): wild broomcorn millet | China, Xinjiang | 2608 |
| 172 | CGRIS (00005818) | China, Yunnan | 2575 |
| T93 | CGRIS (00003368) | China | 2720 |
| T110 | CGRIS (00002527) | China | 2605 |
| T130 | CGRIS (00003056) | China | 3008 |
| T144 | CGRIS (00004228) | China | 2309 |
| T153 | CGRIS (00004704) | China | 2404 |
| T233 | CGRIS (00007455) | China, Shaanxi | 2098 |
| T296 | Field collected: ‘Longmi4’ ^2^ | China, Gansu | 3181 |
| P15 | NPGS (Ames 11693) | India | 2381 |
| P41 | NPGS (PI 170603) | Turkey | 2479 |
| P57 | NPGS (PI 177015) | Turkey | 1550 |
| P79 | NPGS (PI 179392) | Turkey | 1844 |
| P80 | NPGS (PI 180450) | India | 1840 |
| P81 | NPGS (PI 182258) | Turkey | 1479 |
| P128 | NPGS (PI 250979) | Former Serbia and Montenegro | 2034 |
| P130 | NPGS (PI 251388) | Iran | 2325 |
| P140 | NPGS (PI 253955) | Afghanistan | 1658 |
| P142 | NPGS (PI 255736) | Turkey | 1610 |
| P151 | NPGS (PI 269959) | Pakistan | 1412 |
| P175 | NPGS (PI 346934) | Ukraine | 3177 |
| P176 | NPGS (PI 346935) | Former USSR | 2146 |
| P199 | NPGS (PI 380959) | Iran | 1356 |
| P261 | NPGS (PI 463135) | India | 2107 |
| P371 | NPGS (PI 463245) | India | 4347 |
| P602 | NPGS (PI 463476) | India | 4168 |
| P657 | NPGS (PI 531405) | Former USSR | 3476 |
| P658 | NPGS (PI 531406) | Former Czechoslovakia | 3213 |
| P681 | NPGS (PI 536011) | USA | 2879 |
| P697 | NPGS (PI 649382) | USA | 2484 |

Modern broomcorn millet accessions used to generate chloroplast genomes to compare to the Areni-1 millet. Accession numbers are given in parentheses. CGRIS: China Germplasm Resource Information System (https://www.cgris.net/cgris_english.html).

NPGS: The U.S. National Plant Germplasm System (<https://www.ars-grin.gov/npgs/>).

^1^ The nuclear reference genome of this accession was reported by Zou et al (2019)

^2^ The nuclear reference genome of this accession was reported by Shi et al (2019).

**Table S2. Chloroplast reference genomes from NCBI**

| **Species** | **GenBank#** |
| --- | --- |
| *Cenchrus americanus* | KJ490012.1 |
| *Dichanthelium acuminatum* | NC_030623.1 |
| *Panicum capillare* | NC_030493.1 |
| *Panicum sumatrense* | NC_032378.1 |
| *Panicum virgatum* | HQ731441 |
| *Paspalidium geminatum* | KU291476.1 |
| *Setaria italica* | KJ001642.1 |
| *Setaria viridis* | NC_028075.1 |
| *Whiteochloa capillipes* | NC_030618.1 |
| *Sorghum bicolor* | NC_008602.1 |
| *Zea mays* | KF241981.1 |

Chloroplast reference genomes downloaded from NCBI and used with the Areni-1 millet genomes in a Paniceae phylogeny. Maize (*Zea mays*) was included as an outgroup.

**Supplemental Methods**

**Areni-1 millet**

**DNA Extraction and Library Construction**

The extraction of aDNA from the Areni-1 millet grains was performed in the dedicated low-DNA cleanroom at the Australian Centre for Ancient DNA (University of Adelaide), which is physically separated from any post-amplification laboratories and is regularly cleaned with bleach and exposed to UV light. Standard ancient DNA (aDNA) procedures were followed including negative controls for all extractions and amplifications ^1^. Ancient DNA was extracted from individual grains using a previously published protocol ^2^ and described below. Three extraction blanks were assigned identification numbers by LIMS system on entry into sample database (18324, 18335, and 19588) and were processed in an identical manner as the Areni-1 millet grains.

The Areni-1 millet was first washed by placing each grain in a clean 1.5 mL tube containing 100 µL EB Buffer (Qiagen) + 0.05% Tween and then incubating the tube for 5 min in a thermomixer set to 500 RPM at 25°C. The EB buffer was removed and each grain was further washed with 100 µL 100% ethanol in a similar manner. The ethanol was removed, and the grains were allowed to dry in the open tube for 5 min. After drying, each grain was transferred to a screw cap tube containing 5 Zirconia/Silica beads (2.3 mm, Biospec) and ground to powder by agitating the tube in a FastPrep at 6.5 meters/second for 45 seconds. To extract aDNA, 150 µL Plant DNAzol (ThermoFisher) + 2% PVP (Sigma-Aldrich) was added to each tube, which was then shaken for 5 min in a thermomixer for 5 min (500 RMP) at 25°C. One hundred and fifty µL of chloroform was added to each tube and the tubes were vigorously shaken by hand for 5 minutes followed by incubation on a bench top for 15 min. The extraction mixture from each tube was transferred to a fresh 1.7 mL tube and centrifuged at 12,000 g for 10 min. The upper aqueous layer, which contains the aDNA, produced in these extractions was brown in color suggesting the presence of impurities and to remove any substances that may inhibit downstream steps in library construction, the aqueous layer was further purified with MiniElute spin columns (Qiagen) following the manufacturer’s instruction except the final elution was performed with 22 µL EB buffer + 0.05% Tween ^2^.

Extracted aDNA was converted into truncated versions of Illumina double stranded libraries containing dual 7-mer internal barcodes following established protocols ^3-5^. The sequence of all adapters and primers are given in Table 1. The library construction protocol included treatment with the enzyme cocktail USER to partially remove deaminated cytosines ^6^. To generate sufficient library for future shotgun sequencing, plastid enrichment, and hybridization capture, DNA from the *Bst* reactions were taken through two sequential rounds of PCR amplification. For the 1^st^ amplification, the inactivated *Bst* reaction was divided among 8 x 25 µL PCRs containing 2.5 µL 10x High Fidelity PCR Buffer, 5 µL library, 1 µL 50 mM MgSO_4_, 0.5 µL 10 mM dNTPs, 0.5 µL each of 10 µM IS7 and IS8 primers ^4^, 0.1 µL Platinum Taq DNA Polymerase High Fidelity (5 U/μL), and molecular biology grade H_2_O to 25 µL. The PCRs were amplified in a heated-lid thermal cycler programmed as follows: initial denaturation 94ºC for 2 min, 15 cycles at 94ºC for 15 sec, 58ºC for 30 sec, 68ºC for 45 sec, and a final extension at 68ºC for 2 min. Identical PCRs were pooled and the libraries were purified using 1.8x volumes of AMPure XP beads (Beckman) and quantified with a Qubit dsDNA Broad Range assay (ThermoFisher) following the manufacturer’s instructions. Purified library was separated on a 2% TBE agarose gel using electrophoresis and then visualized using GelRed (Biotium) staining and UV illumination. For the 2^nd^ sequential amplification, 60 ng of the 1^st^ amplification was evenly distributed among 16 x 25 µL PCRs containing 2.5 µL 10x High Fidelity PCR Buffer, 1 µL 50 mM MgSO_4_, 0.5 µL 10 mM dNTPs, 0.5 µL each of 10 µM IS7 and IS8 primers, 0.1 µL Platinum Taq DNA Polymerase High Fidelity (5 U/μL), and molecular biology grade H_2_O to 25 µL. Amplification was performed in a heated-lid thermal cycler programmed as follows: initial denaturation 94ºC for 2 min, 9 cycles at 94ºC for 15 sec, 58ºC for 30 sec, 68ºC for 45 sec, and a final extension at 68ºC for 2 min. The library was purified and visualized as before. Libraries generated from this amplification were used for all subsequent steps in this study.

**Table 1: Primers and Oligos for ancient broomcorn millet**

| Ancient Samples Shotgun/cpDNA Enriched Libraries | **IS1_adapter.P5** | **IS2_adapter.P7** |
| --- | --- | --- |
| 18324 | ACACTCTTTCCCTACACGACGCTCTTCCGATCTaagacgt | GTGACTGGAGTTCAGACGTGTGCTCTTCCGATCTgactcgc |
| 18335 | ACACTCTTTCCCTACACGACGCTCTTCCGATCTcacgtcg | GTGACTGGAGTTCAGACGTGTGCTCTTCCGATCTcaggaat |
| 11294a | ACACTCTTTCCCTACACGACGCTCTTCCGATCTgcgtagt | GTGACTGGAGTTCAGACGTGTGCTCTTCCGATCTgcttcca |
| 19558 | ACACTCTTTCCCTACACGACGCTCTTCCGATCTggagtac | GTGACTGGAGTTCAGACGTGTGCTCTTCCGATCTgactgta |
| 11292a | ACACTCTTTCCCTACACGACGCTCTTCCGATCTttctacg | GTGACTGGAGTTCAGACGTGTGCTCTTCCGATCTgactgta |
| 11293a | ACACTCTTTCCCTACACGACGCTCTTCCGATCTaagacgt | GTGACTGGAGTTCAGACGTGTGCTCTTCCGATCTcgccatg |
| 11294b | ACACTCTTTCCCTACACGACGCTCTTCCGATCTcctcgta | GTGACTGGAGTTCAGACGTGTGCTCTTCCGATCTgtggcat |
| 11295a | ACACTCTTTCCCTACACGACGCTCTTCCGATCTcctcgta | GTGACTGGAGTTCAGACGTGTGCTCTTCCGATCTtcgagtg |
|  |  |  |
| Ancient Samples Shotgun Libraries | **IS4_indPCR.P5** | **Indexing Primer** |
| 18324 | AATGATACGGCGACCACCGAGATCTACACTCTTTCCCTACACGACGCTCTT | CAAGCAGAAGACGGCATACGAGAT**atcttgc**GTGACTGGAGTTCAGACGTGT |
| 18335 | AATGATACGGCGACCACCGAGATCTACACTCTTTCCCTACACGACGCTCTT | CAAGCAGAAGACGGCATACGAGAT**atcttgc**GTGACTGGAGTTCAGACGTGT |
| 11294a | AATGATACGGCGACCACCGAGATCTACACTCTTTCCCTACACGACGCTCTT | CAAGCAGAAGACGGCATACGAGAT**catcgag**GTGACTGGAGTTCAGACGTGT |
| 19558 | AATGATACGGCGACCACCGAGATCTACACTCTTTCCCTACACGACGCTCTT | CAAGCAGAAGACGGCATACGAGAT**catcgag**GTGACTGGAGTTCAGACGTGT |
| 11292a | AATGATACGGCGACCACCGAGATCTACACTCTTTCCCTACACGACGCTCTT | CAAGCAGAAGACGGCATACGAGAT**gtaccgg**GTGACTGGAGTTCAGACGTGT |
| 11293a | AATGATACGGCGACCACCGAGATCTACACTCTTTCCCTACACGACGCTCTT | CAAGCAGAAGACGGCATACGAGAT**gtaccgg**GTGACTGGAGTTCAGACGTGT |
| 11294b | AATGATACGGCGACCACCGAGATCTACACTCTTTCCCTACACGACGCTCTT | CAAGCAGAAGACGGCATACGAGAT**gtaccgg**GTGACTGGAGTTCAGACGTGT |
| 11295a | AATGATACGGCGACCACCGAGATCTACACTCTTTCCCTACACGACGCTCTT | CAAGCAGAAGACGGCATACGAGAT**caggtcg**GTGACTGGAGTTCAGACGTGT |
|  |  |  |
| Ancient Samples cpDNA Enriched Libraries | **IS4_indPCR.P5** | **Indexing Primer** |
| 18324 | AATGATACGGCGACCACCGAGATCTACACTCTTTCCCTACACGACGCTCTT | CAAGCAGAAGACGGCATACGAGAT**gtaccgg**GTGACTGGAGTTCAGACGTGT |
| 18335 | AATGATACGGCGACCACCGAGATCTACACTCTTTCCCTACACGACGCTCTT | CAAGCAGAAGACGGCATACGAGAT**gtaccgg**GTGACTGGAGTTCAGACGTGT |
| 11294a | AATGATACGGCGACCACCGAGATCTACACTCTTTCCCTACACGACGCTCTT | CAAGCAGAAGACGGCATACGAGAT**gtaccgg**GTGACTGGAGTTCAGACGTGT |
| 19558 | AATGATACGGCGACCACCGAGATCTACACTCTTTCCCTACACGACGCTCTT | CAAGCAGAAGACGGCATACGAGAT**tctccat**GTGACTGGAGTTCAGACGTGT |
| 11292a | AATGATACGGCGACCACCGAGATCTACACTCTTTCCCTACACGACGCTCTT | CAAGCAGAAGACGGCATACGAGAT**tctccat**GTGACTGGAGTTCAGACGTGT |
| 11293a | AATGATACGGCGACCACCGAGATCTACACTCTTTCCCTACACGACGCTCTT | CAAGCAGAAGACGGCATACGAGAT**tctccat**GTGACTGGAGTTCAGACGTGT |
| 11294b | AATGATACGGCGACCACCGAGATCTACACTCTTTCCCTACACGACGCTCTT | CAAGCAGAAGACGGCATACGAGAT**tctccat**GTGACTGGAGTTCAGACGTGT |
| 11295a | AATGATACGGCGACCACCGAGATCTACACTCTTTCCCTACACGACGCTCTT | CAAGCAGAAGACGGCATACGAGAT**gtaccgg**GTGACTGGAGTTCAGACGTGT |
|  |  |  |
|  | **IS7_short_amp.P5** | **IS8_short_amp.P7** |
|  | ACACTCTTTCCCTACACGAC | GTGACTGGAGTTCAGACGTGT |

Lower case letters are internal adapter barcodes and lowercase letters in bold are i7 adapter indexes.

**Areni-1 Indexed Shotgun Libraries**

Indexed shotgun libraries were generated by distributing 30 ng of amplified library from the previous step in 4 x 25 µL PCR replicates containing: 2.5 µL 10x Taq HiFi Buffer, 1µL 50 mM MgSO4, 0.5 µL, 10 mm dNTPs, 0.5 µL each of 10 µM IS4 primer and 10 µM indexing primer ^5^, 0.1 µL Taq HiFi (5 U/µL), and molecular biology grade H_2_O to 25 µL. Amplification was performed in a heated-lid thermal cycler programmed as follows: initial denaturation at 94°C for 2 min; 6 cycles at 94°C for 10 sec, 58°C for 30 sec, 68°C for 30 sec; and a final extension at 68°C for 2 min. Similar PCRs were pooled, purified, and visualized as before .

**Areni-1 chloroplast hybridization capture**

An RNA probe set to enrich plastid genomes was purchased from Arbor Biosciences (<https://arborbiosci.com>). The probe set was designed using the NCBI references: EU534409 (*Triticum aestivum* mitochondrion, complete genome), NC_002762 (*Triticum aestivum* chloroplast, complete genome), NC_008590 (*Hordeum vulgare* subsp. *vulgare* chloroplast, complete genome) and consisted of 13,524 RNA oligonucleotides that were 80 nt in length and covered target loci with 4x tiling.

To enrich plastid genomes each library was taken through two sequential rounds of hybridization capture ^7^ to maximize the recovery of cpDNA. In each enrichment (1^st^ and 2^nd^), 100-150 ng of amplified library was captured with 1.25 µL of MyBaits probe with the following modification to the manufacturer’s instructions (<http://www.mycroarray.com/pdf/MYbaits-manual-v3.pdf>): RNA oligos were used to block the truncated adapter instead of the MyBaits Block #3 (blocking oligonucleotides for full length adapters) ^8^, SeqCap EZ Developer (Roche) was substituted for the MyBaits Block #1 (human C_0_t -1vDNA), the hybridization temperature was taken through a step-down cycle that was held initially at 65°C for 5 hours, decreased to 60°C for 5 hours, and finished at 55°C for 30 hours, and at the end of the hybridization capture procedure the library was eluted by suspending the streptavidin-coupled beads in 40 µL molecular biology grade H_2_O and heating at 95°C for 5 min.

**Post Enrichment Amplification**

At the start of the study, amplification of captured cpDNA was performed with a set cycle PCR program, which was used with the 1^st^ enrichment of extraction blanks: 18324, 18335 and millet samples: 11294a, 11295a. As the study progressed, a protocol that included a qPCR step to determine the minimum number of PCR cycles to produce sufficient library for downstream procedures was adopted for all further amplification ^9^. Cycle number for each sample at each enrichment is given in Table 2.

**1^st^ Enrichment Amplification**

For the set cycle amplification, eluted cpDNA from each extraction blank/sample was evenly distributed in 8 x 25 µL PCRs containing 2.5 µL 10x High Fidelity PCR Buffer, 5 µL DNA, 1 µL 50 mM MgSO_4_, 0.2 µL 25 mM dNTPs, 0.5 µL each of 10 µM IS7 and IS8 primers, 0.1 µL Platinum Taq DNA Polymerase High Fidelity (5 U/μL), and molecular biology grade H_2_O to 25 µL. Amplification was performed in a heated-lid thermal cycler programmed as follows: initial denaturation 94ºC for 2 min, 18 cycles at 94ºC for 15 sec, 58ºC for 30 sec, 68ºC for 45 sec, and a final extension at 68ºC for 2 min.

In the variable cycle amplifications, 1 µL of the eluted cpDNA from each extraction blank/sample was quantified to determine the proper cycle number ^9^. The remaining library was distributed among 8 x 25 µL PCRs containing 2.5 µL 10x High Fidelity PCR Buffer, 4.88 µL DNA, 1 µL 50 mM MgSO_4_, 0.2 µL 25 mM dNTPs, 0.5 µL each of 10 µM IS7 and IS8 primers, 0.1 µL Platinum Taq DNA Polymerase High Fidelity (5 U/μL), and molecular biology grade H_2_O to 25 µL. Amplification was performed in a heated-lid thermal cycler programmed as follows: initial denaturation 94ºC for 2 min, *X* cycles (Table 2) at 94ºC for 15 sec, 58ºC for 30 sec, 68ºC for 45 sec, and a final extension at 68ºC for 2 min. Identical PCRs were pooled then quantified and visualized as before.

**2^nd^ Enrichment Amplification**

One µL of the eluted cpDNA was quantified with qPCR as before. The remaining DNA from each sample was distributed among 8 x 25 µL PCRs containing 2.5 µL 10x Gold PCR Buffer, 4.88 µL DNA, 2 µL 2.5 mM dNTPs, 1.5 µL 25 mM MgCl_2,_ 0.5 µL each of 10 µM IS4 and indexing primers ^4^, 0.125 µL AmpliTaq Gold DNA Polymerase, and molecular biology grade H_2_O to 25 µL and amplified in a heated-lid thermal cycler programmed as follows: initial denaturation 94ºC for 2 min, *X* cycles (Table 2) at 94ºC for 15 sec, 58ºC for 30 sec, 68ºC for 45 sec, and a final extension at 68ºC for 2 min. Identical PCRs were pooled then quantified and visualized as before.

**Table 2: PCR cycle number**

| Sample | # of Cycles |
| --- | --- |
|  |  |
| *1^st^ Enrichment* |  |
| 19558 (Extraction Blank) | 24 |
| 11292a | 14 |
| 11293a | 19 |
| 11294b | 22 |
| 18335 (Extraction Blank) | **18** |
| 11294a | **18** |
| 18324 (Extraction Blank) | **18** |
| 11295a | **18** |
|  |  |
| *2^nd^ Enrichment* |  |
| 19558 (Extraction Blank) | 14 |
| 11292a | 9 |
| 11293a | 13 |
| 11294b | 14 |
| 18335 (Extraction Blank) | 22 |
| 11294a | 13 |
| 18324 (Extraction Blank) | 24 |
| 11295a | 9 |

Amplification of the cpDNA enriched libraries was performed with two different protocol over the course of the study. Initially, post-enrichment amplification was performed with a set cycle PCR program (samples in bold). Subsequently, a protocol that quantified each library with qPCR to determine the minimal number of cycles to produce sufficient DNA for downstream procedures was used for all other amplifications (samples in standard text) ^9^.

**Areni-1 high-throughput sequencing**

Shotgun and cpDNA enriched libraries were quantified on 4200 TapeStation using a D1000 ScreenTape assay (Agilent). Shotgun libraries were pooled in equimolar amounts and sequenced on a NextSeq 500 platform except for extraction blank 18324 which was sequenced on a MiSeq with samples from other studies. The shotgun sequencing was performed the ACRF Cancer Genomics Facility (Adelaide, Australia) using Mid-Output 2 x 150 bp paired-end (300 cycles) chemistry for the NextSeq run and 2 x 150 bp paired-end (300 cycles) chemistry for the MiSeq run. The cpDNA enriched libraries were pooled and sequenced at the Kinghorn Centre for Clinical Genomics (Sydney, Australia) with a single lane of a HiSeq X Ten flow-cell using 2 x 150 bp paired-end (300 cycles) chemistry.

**References**

1 Cooper, A. & Poinar, H. N. Ancient DNA: Do it right or not at ALL. *Science* **289**, 1139-1139 (2000).

2 Richards, S. M. *et al.* Low-cost cross-taxon enrichment of mitochondrial DNA using in-house synthesised RNA probes. *PLOS ONE* **14**, e0209499, doi:10.1371/journal.pone.0209499 (2019).

3 Llamas, B. *et al.* Ancient mitochondrial DNA provides high-resolution time scale of the peopling of the Americas. *Science Advances* **2**, doi:10.1126/sciadv.1501385 (2016).

4 Meyer, M. & Kircher, M. Illumina Sequencing Library Preparation for Highly Multiplexed Target Capture and Sequencing. *Cold Spring Harbor Protocols* **2010**, pdb.prot5448, doi:10.1101/pdb.prot5448 (2010).

5 Knapp, M., Stiller, M. & Meyer, M. Generating barcoded libraries for multiplex high-throughput sequencing. *Methods in Molecular Biology* **840**, 155-170 (2012).

6 Rohland, N., Harney, E., Mallick, S., Nordenfelt, S. & Reich, D. Partial uracil–DNA–glycosylase treatment for screening of ancient DNA. *Philosophical Transactions of the Royal Society of London B: Biological Sciences* **370**, doi:10.1098/rstb.2013.0624 (2015).

7 Fu, Q. M. *et al.* DNA analysis of an early modern human from Tianyuan Cave, China. *Proceedings of the National Academy of Sciences of the United States of America* **110**, 2223-2227, doi:10.1073/pnas.1221359110 (2013).

8 Fehren-Schmitz, L. *et al.* A Re-Appraisal of the Early Andean Human Remains from Lauricocha in Peru. *PLoS ONE* **10**, e0127141, doi:10.1371/journal.pone.0127141 (2015).

9 Carøe, C. *et al.* Single-tube library preparation for degraded DNA. *Methods in Ecology and Evolution*, n/a-n/a, doi:10.1111/2041-210X.12871 (2017).
